# Ischemic Stroke: Do Not Forget Lyme Neuroborreliosis

**DOI:** 10.1155/2018/1720725

**Published:** 2018-04-01

**Authors:** Gabriela Moreno Legast, Armin Schnider, Nicolas Nicastro

**Affiliations:** Division of Neurorehabilitation, Geneva University Hospitals, Geneva, Switzerland

## Abstract

Lyme neuroborreliosis is a rare cause of ischemic stroke; it has only been described in case reports and mostly in Europe. Diagnostic criteria have been proposed for Lyme neuroborreliosis but the association with a cerebral ischemic presentation is not always straightforward. We here describe the case of an 83-year-old man for whom we strongly suspect Lyme neuroborreliosis as the etiology of his stroke. This case reminds us of the importance of a thorough history taking (i.e., tick bite) and to perform the adequate ancillary tests accordingly (lumbar puncture) so as to propose validated treatment.

## 1. Introduction

Lyme disease is the most common tick-borne illness in Europe and the United States [1]. It is caused by the spirochete* Borrelia burgdorferi* sensu lato, and its incidence has shown a rise in the last few years.

11% of the patients with* B*.* burgdorferi* infection present with neurological symptoms [1], lymphocytic meningitis, cranial neuritis, and radiculoneuritis being the most common presentations. Lyme neuroborreliosis (LNB) can rarely present as a stroke due to cerebral vasculitis. We here present a patient with ischemic stroke whose suspected etiology is LNB.

## 2. Case Presentation

**Figure 1:**
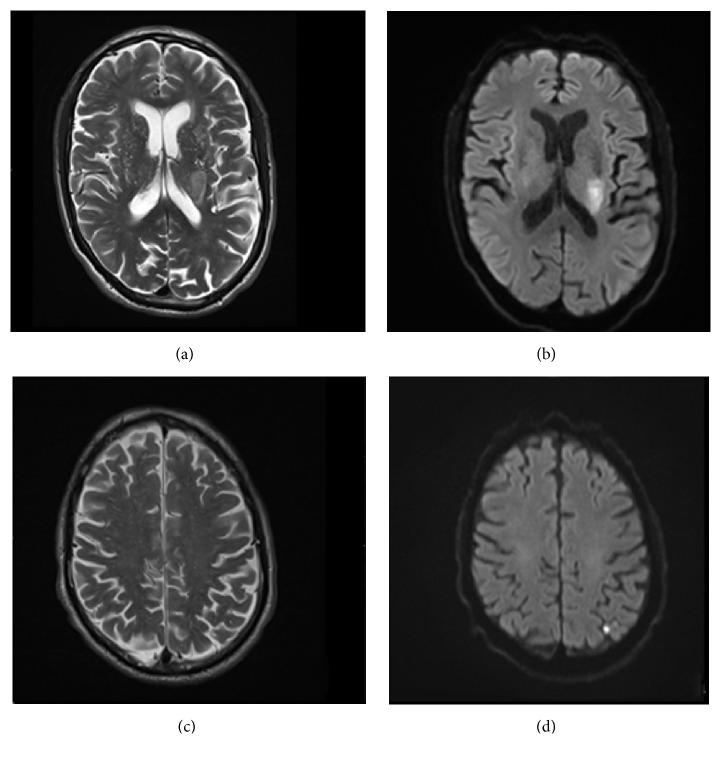
Brain MRI showing left corona radiata and parietal ischemic lesion on T2-weighted MRI (a, c) and diffusion-weighted imaging (DWI, (b), (d)).

An 83-year-old man presented to the emergency room with right-sided weakness and speech difficulties evolving for 24 hours. He did not report any neurological or cardiovascular medical history. Neurological examination confirmed severe right hemiparesis and dysarthria. Brain CT scan showed a hypodensity in the left corona radiata. Angiographic sequences found a left M2 segment subocclusive stenosis. MRI studies confirmed a recent ischemic lesion in the left corona radiata as well as a small left parietal lesion (Figure 1). Investigations did not reveal any argument for large vessel disease or cardiac source and the patient had no history of hypertension. Homocysteinemia was normal as well as lipid profile; lupus anticoagulant was positive for this patient known neither for venous or arterial thrombosis nor for autoimmune disease. Infectious screening was negative for HIV, HSV2, influenza, and RSV. Patient was immune for EBV, CMV, and HSV1. VDRL was negative. Lyme serology was positive on both ELISA (IgM 1.5 and IgG 7.3) and Western blot. Medical history was then reassessed; the patient described multiple tick bites in the past few years, without noticing any skin lesion evoking erythema chronicum migrans. He also recalled having paresthesia with tingling in his hands and feet 2 years ago during 4 months. A lumbar puncture yielded a normal cell count (1 M/L leukocytes and 89 M/L erythrocytes), but elevated protein level at 0.71 g/l, elevated albumin quotient (8.27), and elevated IgG synthesis in the CSF (46 mg/l) as well as increased specific CSF/serum antibody index for Lyme IgG at 27.5. There were no oligoclonal bands. Considering the possibility of a stage 3 LNB with cerebrovascular complications, antibiotic treatment with intravenous Ceftriaxone 2 g per day was administered for 14 days. Antiphospholipid antibodies were retested 6 weeks later and were all negative. Lyme serology was reassessed two months after antibiotic treatment and showed normal blood IgM titers (0.9). The patient slowly improved and was discharged a few weeks later. He could walk with a cane but still had a severe right arm paresis.

## 3. Discussion

LNB is a rare cause of stroke; it has been mostly described in European case reports or case studies [2–4]. Nowadays there is no evidence for testing Lyme disease in every patient with an ischemic stroke. However, this can be considered in the following situations: when a patient comes from an endemic area, with a history of* B. burgdorferi* infection, when no other evident cause of stroke is found, and in case of multiterritorial strokes or radiological signs of vasculitis [2, 3]. Regarding our patient, investigations for infectious or autoimmune diseases were performed because of a lupus anticoagulant antibody. Antiphospholipid antibodies are associated with different infectious diseases [5]. Even though this has been poorly described in Lyme disease, Greco et al. showed a frequent association in patients with chronic Lyme disease [6], possibly explained by molecular mimicry [7].

In the presence of a lupus anticoagulant antibody and no obvious risk factors like hypertension, we considered further investigations for Lyme disease, the more so as our patient lived in an endemic area (Western Switzerland) and had multiple tick bites in the past few months. According to the European Federation of Neurological Societies (EFNS) diagnostic criteria, our patient had a possible but not definite LNB because of absent CSF pleocytosis [8]. Besides, the presence of IgM Lyme antibodies confirms a recent infection but does not prove it was the cause of the present stroke. This case underlines the importance of a detailed medical history in order to guide investigations.

As our patient showed important residual symptoms with functional limitation (walking difficulty, right arm paresis), this case reminds us that, even after standard pharmacological treatment, LNB can lead to long-term sequels [9].
